# Unsupervised Clustering of Cell Populations in Germinal Centers Using Multiplexed Immunofluorescence

**DOI:** 10.3390/biology14050530

**Published:** 2025-05-11

**Authors:** Simon Burgermeister, Michail Orfanakis, Spiros Georgakis, Cloe Brenna, Helen Lindsay, Craig Fenwick, Giuseppe Pantaleo, Raphael Gottardo, Constantinos Petrovas

**Affiliations:** 1 Department of Laboratory Medicine and Pathology, Institute of Pathology, Lausanne University Hospital, University of Lausanne, CH-1011 Lausanne, Switzerland; 2 Biomedical Data Science Center, Lausanne University Hospital, Lausanne University, CH-1011 Lausanne, Switzerland; helen.lindsay@chuv.ch (H.L.); raphael.gottardo@chuv.ch (R.G.); 3 Service of Immunology and Allergy, Department of Medicine, Lausanne University Hospital, Lausanne University, CH-1011 Lausanne, Switzerland; giuseppe.pantaleo@chuv.ch

**Keywords:** multiplex immunofluorescence, unsupervised clustering, germinal centers

## Abstract

Follicles (Fs)/Germinal Centers (GCs) in tonsils and lymph nodes are dynamic microenvironments where diverse immune cell populations interact for the development of antibody responses against pathogens. The accurate in situ phenotypic analysis of these immune cells is a prerequisite for the comphehensive understanding of GC development. In this study, we explore unsupervised clustering approaches for distinguishing cell populations within F/GCs using marker expression data. We evaluate multiple clustering algorithms and find that k-means clustering provides the most effective separation of distinct cell subsets. Additionally, we investigate the predictive potential of common GC markers (CD3, CD4, CD20 and BCL6) for PD-1 expression, an important immune checkpoint regulator. Our analysis demonstrates that PD-1 expression can be reliably inferred using these markers, suggesting potential applications for automated cell classification in immunological studies. This approach enhances our ability to analyze immune cell heterogeneity and may contribute to improved understanding of GC dynamics in health and disease. Our findings support the use of computational clustering for high-dimensional immune profiling.

## Simple Summary

The follicular/germinal center immune reactivity determine the development of pathogen and immunogen induced antibody responses while is a critical factor for the pathogenesis of chronic diseases like HIV and certain lymphomas. Delineation of the follicular/germinal center immune landscape provides critical information for the understanding of this immune reactivity. To this end, the development of imaging assays and computational tools for imaging data analysis is of great importance. Our data support the use of computational clustering of cell types defined by multiplex imaging as well as the application of complementary methodologies for the high-dimensional characterization of follicular/germinal center immune landscape.

## 1. Introduction

Multiplex immunofluorescence (mIF) is a technique that allows the simultaneous detection and quantification of multiple protein markers within a single tissue section. This method improves traditional immunohistochemistry (IHC) by overcoming its limitation of labeling only one marker per tissue section, thus providing a more comprehensive analysis of cell composition, cellular functions, and cell-to-cell interactions [1]. Multiplex immunofluorescence allows for the simultaneous visualization of multiple biomarkers within a single tissue section at the cellular level, providing a comprehensive view of the tumor microenvironment, cell phenotypes, and other complex tissue architectures. This technique is crucial to understanding the interactions between different cell types and the spatial organization of tissues, which are essential for accurate disease diagnosis and prognosis. However, a major bottleneck in miF data analysis is to accurately assign cell types based on the expression of measured markers at the cellular level [2]. In the context, the use of unsupervised clustering in multiplex immunofluorescence is increasingly important for pathology research and clinical applications due to its ability to enhance the characterization of complex cellular phenotypes [1,3]. Unsupervised clustering algorithms could play a pivotal role in the analysis of high-dimensional data generated by such methods. These algorithms can identify patterns and structures within the data without prior labeling or knowledge of the data, allowing the discovery of novel tissue architectures and cellular interactions that might not be evident through traditional analysis methods. This approach is particularly valuable in pathology, where understanding the spatial distribution and interaction of cells can lead to better insights into disease mechanisms and the development of targeted therapies. They have been shown to be effective in segregating different cell populations on flow cytometry data [4] as well as spatial mIF [5]. Furthermore, unsupervised clustering facilitates the integration of multiplex immunofluorescence data with other high-throughput techniques, such as spatial transcriptomics, to provide a more holistic view of tissue biology. This integration can improve the identification of microanatomical domains and enhance the predictive power of diagnostic models, ultimately contributing to more personalized and effective clinical interventions. As the field of digital pathology continues to evolve, the optimization and standardization of these analytical techniques will be essential for their routine application in clinical settings.

The development of humoral responses against a pathogen or immunogen requires the coordinated function of several cell types (stromal cells, innate and adaptive immune cell types) [6] as well as soluble mediators [7]. Follicular helper CD4 T cells (Tfh) and germinal center (GC) B cells are the main immune cell types mediating the development of antigen specific antibodies [8]. Tonsils represent a prototype lymphoid organ for studying follicular/germinal center immune dynamics. Furthermore, lymphoid organs are important anatomical sites for chronic diseases like lymphomas [9] and HIV infection [10]. Therefore, the comprehensive analysis of F/GC immune dynamics is of great interest for the understanding of disease pathogenesis and the development of novel therapeutic targets. We used mIF data from human tonsils to validate unsupervised methods for the clustering of relevant cell types/biomarkers.

## 2. Materials and Methods

### 2.1. Human Material-Ethical Approval

The tissue samples used in this study were obtained from (i) the archives of the Institute of Pathology of Lausanne University Hospital, Switzerland (cancer-free, HIV-free reactive LNs) and their use was approved by the Ethical Committee of the Canton de Vaud, Switzerland (2021-01161) (Table A1) and (ii) from the Hospital de l’Enfance of Lausanne (tonsils were obtained from anonymized children during routine tonsillectomy) and their use was approved by the Canton de Vaud-CER-VD, Switzerland (PB_2016-02436 (201/11)). Tissues from participants with written consent were used while all procedures were in accordance with the Declaration of Helsinki.

### 2.2. Tissue Processing and Staining

Fresh tissues were fixed in formalin overnight as soon as possible after biopsy and processed for the preparation of formalin-fixed, paraffin-embedded (FFPE) blocks using standard procedures. The blocks were sequentially cut into 4–5 μm sections and prepared on Superfrost glass slides (Thermo Scientific, Waltham, MA, USA, Ref. J1800AMNZ), dried overnight and stored at 4 °C. Before staining, the slides were heated on a metal hotplate (Stretching Table, Medite, Burgdorf, OTS 40.2025, Ref. 9064740715) at 65 °C for 30 min. Tissue sections were stained with titrated antibodies using a Ventana Discovery Ultra Autostainer (Roche Diagnostics, Ventana Medical Systems, Tucson, AZ, USA). Tissues were deparaffinized, hydrated and the protein epitopes were retrieved by applying the standard Ventana Discovery’s protocols. Before all antibody incubation steps, tissues were blocked using Antibody Diluent/Block from Akoya (ARD1001EA, Akoya Biosciences, Marlborough, MA, USA). The cycling staining/imaging approach we developed consisted of two staining cycles. During the first staining cycle, unconjugated and conjugated primary antibodies coupled with Alexa Fluor dyes were used (CD3, CD4, PD1, CD20, Ki67, CD57) (Table A2). Both unconjugated and conjugated antibodies were diluted in Antibody Diluent/Block and incubated sequentially for 90 min at room temperature (RT). Alexa Fluor-conjugated antibodies were diluted in Antibody Diluent/Block and incubated for 45 min at RT. To avoid any unwanted secondary antibody binding to conjugated antibodies we started by incubating unconjugated antibodies, followed by secondary antibodies and lastly with the conjugated antibodies. The samples were then counterstained with SYTO45 (1/10,000 dilution in TBS-T, CatNo 10297192, ThermoFischer Scientific for 40 min, rinsed in soapy water and mounted using DAKO mounting medium (Dako/Agilent, Santa Clara, CA, USA, Ref. S302380-2). After imaging the first cycle, slide coverslips were carefully de-mounted using warm ddH_2_O, and slides were washed briefly in PBS. Then, the first cycle’s antibodies were stripped off using Cell Conditioning Solution (CC2, 950-223, Roche Diagnostics) for 10 min at 100 °C. After the stripping step, slides were washed 2× (ca 10s) in ddH_2_O and 1× for 5 min in PBS and the second cycle of staining followed. During the second cycle, an Opal dye (Opal 7-color Automation IHC kit, from Akoya, Ref. NEL821001KT T) was used to amplify the signal of second-cycle primary antibodies (Bcl6). More specifically, tissue sections were sequentially subjected to antibody blocking, staining with primary antibodies, incubation with secondary HRP-conjugated antibodies (DISCOVERY OmniMap anti-Ms HRP/760-4310, DISCOVERY OmniMap anti-Rb HRP/760-4311) for 16 min, detection with optimized fluorescent Opal tyramide signal amplification (TSA) dyes and repeated antibody denaturation cycles. The samples were then counterstained with SYTO40 (1/10,000 dilution in TBS-T, CatNo, ThermoFischer Scientific for 40 min rinsed in soapy water and mounted using DAKO mounting medium (Dako/Agilent, Santa Clara, CA, USA, Ref. S302380-2). Alternatively, tonsillar tissue sections were stained using the Ventana system and titrated primary antibodies (same clones as above) and appropriate secondary HRP-labeled antibodies with repeated cycles of antibody denaturation (Table A2). Optimized fluorescent Opal tyramide signal amplification (TSA) dyes were used for the detection of all target proteins. For the cell nuclei visualization, the sections were counterstained with Spectral DAPI. Following staining, the sections were rinsed in water with soap and mounted using DAKO mounting medium from Dako/Agilent, Santa Clara.

### 2.3. Data Acquisition

Images were acquired using a Leica Stellaris 8 SP8 confocal system (Leica Microsystems CMS GmbH, Mannheim, Germany), equipped with Leica Application Suite X (LAS-X)-4.6.1.27508 software, at 512 × 512-pixel density, 0.75× optical zoom and a z-step of 1 μm using a 20× objective (NA). At least 70% of each section was imaged, to ensure an accurate representation and minimize selection bias. Tissues stained with a single antibody fluorophore combination were used to create a compensation matrix via the Leica LAS-AF Channel Dye Separation module (Leica Microsystems, Leica Microsystems CMS GmbH, Mannheim, Germany), which was used to correct fluorophore spillover (when present), as per the user’s manual. When the dye separation results were not optimal, the manual LAS-AF Channel Dye Separation module was employed. Alternatively, Multispectral images (MSI) were acquired using the Vectra Polaris 1.0 imaging system (Akoya Biosciences, Marlborough, MA, USA) at a resolution of 5 μm/pixel (20×).

### 2.4. Image Alignment and Registration-Cell Segmentation

The images generated during the two cycles were aligned using SimpleITK [11] as an Imaris extension (Imaris software version 9.9.0, Biplane. To facilitate registration, we utilized one common channel present in both imaging cycles (SYTO). After successful alignment, the Surface Creation module of Imaris was used to generate 3-dimensional segmented surfaces (based on the nuclear signal) of spillover-corrected images. The segmented cells were then processed with the filtering Imaris module using different combinations of filtering types based on the mean and median intensities of channels to be able to exclude artifacts that are characterized by uniform staining across the segmented area. Areas with uniform staining were excluded among the different tissues. Data generated, such as average voxel intensities for all channels and the volume and sphericity of the 3-dimensional surfaces, were exported.

### 2.5. Histocytometry

Acquired images were further processed (Imaris, version 9.8, Oxford Instruments, Abingdon, UK for confocal images and Phenochart 1.0.12 and InForm, version 2.4.8 software (Akoya) for Polaris images) for cell segmentation as previously described [12,13]. A csv file report was generated, containing the spatial coordinates (X, Y) of each segmented cell, along with their mean intensity for each fluorophore used. After converting it to FACS file (FSC) format the data were uploaded to the FlowJo 10 software for further analysis using HistoFlowCytometry [13,14]. Data are reported as frequency of total imaged cells per follicular area.

### 2.6. Marker Spillover Correction

To evaluate if spillover played a role in the cell poppulation identification, we used REDSEA [15] using probability maps generated by Ilastik [16]. SYTO was specified as the nuclear marker, and parameters set as for the MIBI example data. Spillover correction was performed for CD20 and CD3. We used Ilastik v1.4 to classify pixels as nuclear or non-nuclear based on SYTO expression. All available features provided by Ilastik were used for training, with sigma values of 0.3 and 0.7 selected. The classification was exported as a probability map tiff image for the nuclear class.

### 2.7. Cell Phenotyping with Mass Cytometry (CyTOF)

Tonsillar single cell suspensions were used for the phenotypic characterization of relevant immune cell types by CyTOF. Cryopreserved tonsil cells were thawed and resuspended in complete RPMI medium (10% heat inactivated FBS, Life Technologies, Gibco (Waltham, MA, USA), 100 IU/mL penicillin, and 100 μg/mL streptomycin, BioConcept) and rested for 6 h at 37 °C with 5 U/mL Benzonase (Thermo Fisher, Waltham, MA, USA) to minimize cell aggregation. Cells were washed (0.5% BSA-PBS, Sigma, St. Louis, MO, USA) and incubated for 20 min with a 50 μL antibody cocktail of cell surface titrated metal-conjugated antibodies (Table A3). Cell-IDTM-103 Rh Intercalator at a final concentration of 1 μM was used for the cell viability staining. Following a washing and fixation (10 min, RT with 2.4% paraformaldehyde (PFA, Thermo Fisher) step, cells were permeabilized (45 min at 4 °C with the Foxp3 Fixation/Permeabilization kit, eBioscience, Waltham, MA, USA), washed and stained (30 min, 4 °C) with a 50 μL cocktail of intracellular metal conjugated antibodies. For TCF1 assessment, antibody is conjugated with phycoerythrin fluorochrome (Clone 7F11A10, Cat. N. 655208, Biolegend, San Diego, CA, USA) and then detected by metal conjugated anti-PE (145Nd, Standard Biotools, Billerica, MA, USA). Cells were washed and fixed for 10 min at RT with 2.4% PFA. Total cells were identified by DNA intercalation (1 mM Cell-ID Intercalator, Standard Biotools) in 1% PFA and 0.3% saponin (Sigma) at 4 °C, overnight. For CyTOF analysis, cells were washed (×3, MilliQ water, Burlington, MA, USA) and resuspended at 0.5 × 10^6^ cells/ml in 0.1% EQ™ Four Element Calibration Beads solution (Standard Biotools). Data were acquired using a Helios mass cytometer instrument (Standard Biotools), using a flow rate of 0.030 mL/min and an event rate of 300 cells/s. Flow cytometry standard (FCS) files were normalized to EQ Four Element calibration beads using CyTOF software, version 7.0.8493. For conventional cytometric analysis of immune cell populations, FCS files were imported into Cytobank data analysis software for processing with more in-depth analysis performed in R using the OpenCyto and cytofkit packages.

### 2.8. Bioinformatic Analysis (Tonsilar scRNA Data)

To examine gene expression in doublets in single cell RNA-seq (scRNA-seq) data, we downloaded the unfiltered counts tables from an atlas of tonsil scRNA-seq [17]. We selected cells from 9 donors processed at the same hospital. For each sample, we choose “gates” by manually inspecting a density scatterplot of counts for CD3D versus MS4A1 (the gene encoding the CD20). Using these gates, we selected CD3D- and MS4A1-positive “doublets”, CD3D-positive MS4A1-negative (putative T-cells), and MS4A1-positive CD3D-negative (putative B-cells). With the selected B- and T-cell populations as input, we used scDblFinder v 1.18.0 [18] to generate three times as many artificial B-cell-T-cell doublets as there were cells. We then randomly subsampled the artificial doublets to 500 cells per sample. Using Seurat v5.2.1 [19], we performed differential expression analyses between the real and artificial doublets using the default Wilcoxon test. We also examined average gene expression in the members of the Gene Ontology category “leukocyte cell adhesion” (GO:0007159). Analyses were performed using R version 4.4.2.

### 2.9. Statistical Analysis and Cell Population Clustering

We use K-Means, Affinity Propagation, Mean shift Clustering, Agglomerative Clustering, Density-Based Spatial Clustering, OPTICS Clustering, and Birch Clustering and evaluate their performance using Silhouette, Calinski-Harabasz and Davies-Bouldin. The computations were performed using the numpy [20], Scikit-learn [21] and pycaret [22] libraries with python version 3.11.7 Figures were created using the matplotlib [23], seaborn [24] and Vega-Altair [25] libraries. For clustering algorithms that require the number of clusters as input, this number was set to 4. Other inputs were kept to default values. For marker prediction, we used the pycaret classification class with all possible default models including boosting algorithms, tree-based models, and linear models. The model was trained on data from one lymph node and one tonsil selected randomly and evaluated on the remaining data. The models were selected based on AUC to avoid biases introduced by class imbalance. Scaling of cell marker expression was performed using the StandardScaler class from Scikit-learn. The models were interpreted using Shapley Additive exPlanations (SHARP) [26].

## 3. Results

### 3.1. Unsupervised Clustering Analysis of Human Tonsillar Germinal Center Immune Cell Types

**Figure 1:**
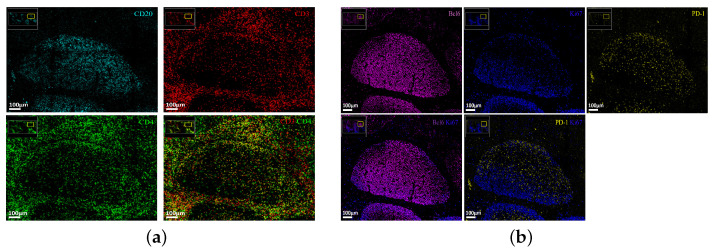
Compartmentalization of immune cell types in tonsillar follicular areas. (**a**) Representative confocal immunofluorescence images (mIFs) showing the expression of CD20 (cyan), a B cell marker, CD3 (red) and CD4 (green), T cell markers and a merged CD3/CD4 image, centered in a F/GC area. (**b**) Representative mIF images showing the expression of Bcl6 (magenta), Ki67 (blue), PD1 (yellow), as well as merged images (Bcl6/Ki67, Ki67/PD-1) in a human tonsillar follicular area.

Human tonsils represent a ‘prototype’ lymphoid organ for the study of F/GC immune landscape [27]. We started our investigation by staining tonsillar tissues with a ‘cycling’ mIF (8-plex, Table A2) assay using antibodies against molecules expressed by the main F/GC immune cell types (anti-CD3 and anti-CD4, T cell markers and anti-CD20, a B cell marker) (Figure 1a). Further, tissues were stained for PD-1, a prototype marker for Tfh cells [28], Ki67, a proliferation marker and Bcl-6 a master regulator of both Tfh and GC B cells [29] (Figure 1b). Although Bcl-6 expression was widely distributed across the follicular area, the expression of Ki67 was more polarized with some areas highly enriched in Ki67^high^ cells (Figure 1b). A distinct pattern for localizing PD-1^high^ cells in areas less populated by Ki67^high^ cells was consistently found (Figure 1b).

**Figure 2:**
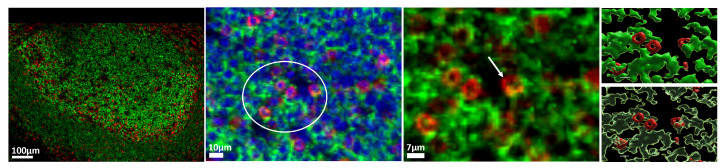
Representative confocal images showing the expression of CD3 (red), CD20 (green), and nuclear (blue) in a tonsillar follicle (scale bar: 70 μm) (left panel) as well as zoomed areas (scale bar: 10 μm and 5 μm, middle panel). The interconnected/fused membranes between cells are shown too (right panels). White arrow points to a CD3/CD20 ‘conjugate’ in a zoomed area (white circle). Imaris modules were used for the generation of ‘isothermic’ surfaces for the visualization of the CD3 (red)/CD20 (green) membranes.

Cellular events showing a ‘co-localization’ of CD3 and CD20 were evident across the follicular areas analyzed (Figure 2), presumably depicting areas where nearby cells have interconnected membrane structures.

**Figure 3:**
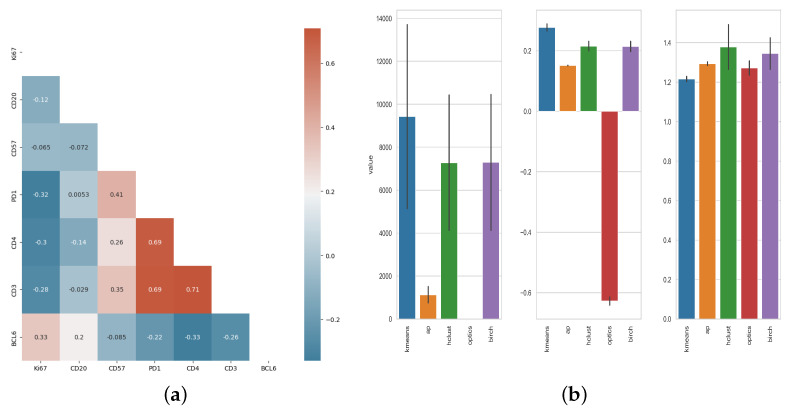
(**a**) Calculated correlations between markers used for the characterization of tonsilar follicular/GC immune cell types. Correlation is represented through Pearson’s R. (**b**) Clustering scores for tonsils germinal centers cell populations. Silhouette & Calinski-Harabasz: higher values indicate a better clustering result. Davies-Bouldin, lower values indicate a better clustering result. Bar indicate standard deviation between GC areas.

For each segmented cellular event, the position (X, Y) coordinates along with the raw intensities of the markers used were extracted and used for downstream analysis. A consistent correlation among PD1, CD3, and CD4 was found, with Pearson’s R coefficients ranging from 0.69 to 0.71 (Figure 3a), while Ki67 and CD20 had the weakest correlation with other markers (Pearson’s R range: [−0.3, 0.33] and [−0.14, 0.2] respectively). Then, different clustering algorithms were applied for further unsupervised analysis of the imaging data. The performance of each algorithm was evaluated based on its score for Silhouette, Calinski-Harabasz and Davies-Bouldin profiles (Figure 3b).

The K-mean algorithm provided the best differentiation of the data based on Sihouette profile, Davies-Bouldin and Calinski-Harabasz index, with Silhouette profiles averaging 0.28 (range: 0.27–0.29). The other metrics used for evaluations of the cell clusters also highlighted the superiority of the K-means algorithm with higher Calinski-Harabasz and lower Davies-Bouldin respectively (Figure 3b), in line with the aforementioned mIF (Figure 1). Specifically CD4 was found to be co-expressed with CD3 while a similar profile was found for PD1 too (Figure 3a). Bcl6 was found mainly co-expressed with CD20^high^ cells with or without expression of Ki67. Details about the different clusters and the number of cells in each group per tonsil sample are listed in Table 1.

**Figure 4:**
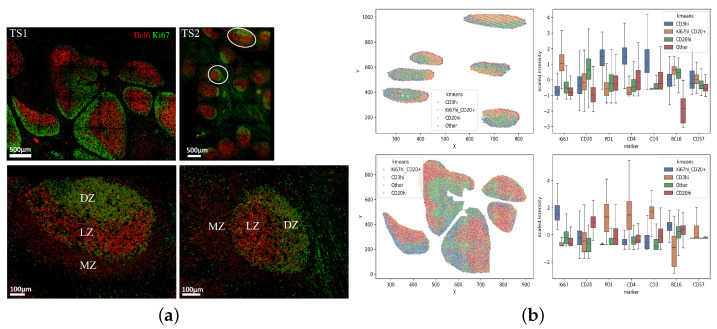
Identification of immune cell clusters in tonsillar follicular areas (cycling mIF). (**a**) Distinct follicular/GC areas in tonsilar follicles can be identified based on the expression of CD20 (red) and Ki67 (green). Areas enriched in KI67 positive CD20 cells represent the Dark Zone (DZ), while the Light Zone (LZ) area is less populated by Ki67-positive B cells. The Mantle Zone is characterized by the expression of CD20 and the absence of proliferating, Ki67-positive Data from two tonsils (upper panel) are shown. Two zoomed follicular areas (white circles) are shown in the lower panel. (**b**) F/GCs from two tonsils were used for our analysis. The digital representation of identified clusters, using the Kmeans algorithm, are shown for both tonsils as well as the relative expression of individual analyzed markers in each immune cell cluster.

Despite the variation in the expression of a given marker within a specific cell type, the resulting marker expression signatures are consistent with known cell types found in healthy germinal centers. Specifically, our approach correctly identifies a population with high prevalence of Ki67^high^CD20^high^ events, enriched in the Dark Zone as well as Ki67^low^CD20^high^ B cells, enriched in the Light Zone [30] (Figure 4a). With respect to CD3 expression, we observed a low but measurable ‘co-expression’ with CD20 (Figure 4b), in line with the aforementioned mIF. Again, CD4 was found to be co-expressed with CD3 while a similar profile was found for PD-1 too (Figure 4b). Bcl6 was found to be mainly co-expressed with CD20^high^ cells with or without Ki67 expression (Figure 4b). Despite the noise and marker spillage, our unsupervised clustering analysis can accurately identify follicular/germinal center immune cell types.

**Figure 5:**
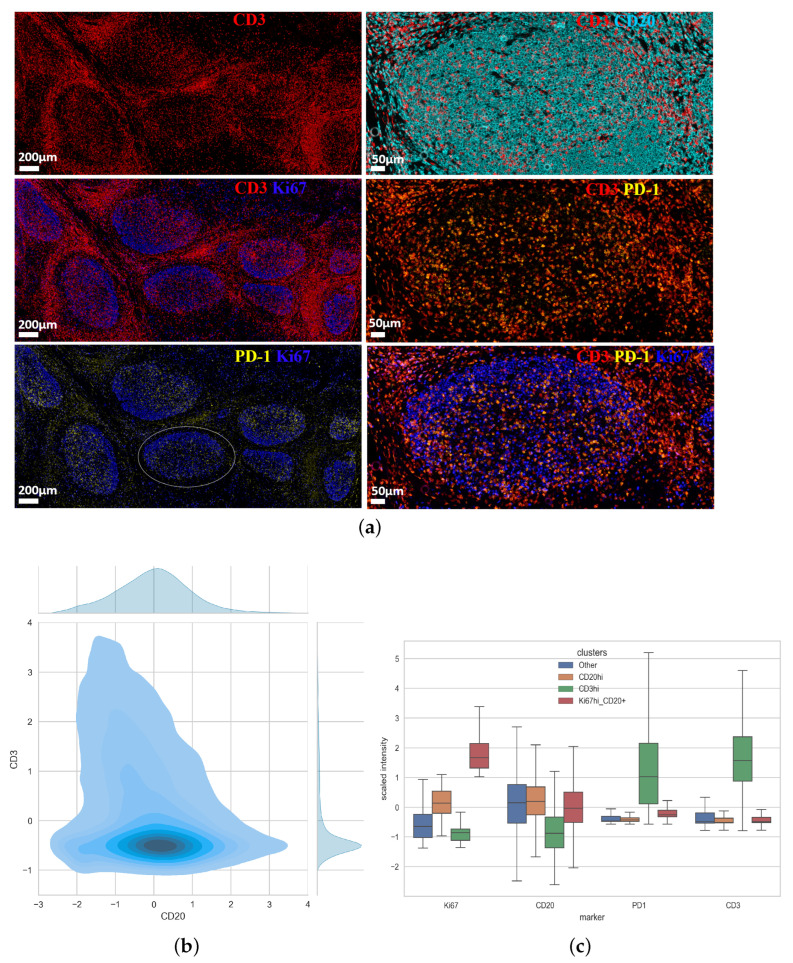
Identification of immune cell clusters in tonsillar follicular areas (Polaris mIF). (**a**) Representative images showing the expression of CD3 (red), CD20 (cyan), Ki67 (blue) and PD-1 (yellow) in tonsillar follicles (scale bar: 200 μm). A zoomed follicular area (white circle, scale bar 50) is shown on the right panels too. (**b**) A 2D plot representation of CD3 and CD20 expression in one tonsillar follicular areas. (**c**) The Kmeans algorithm was applied and the relative expression of individual analyzed markers in each immune cell cluster is shown.

Then, we sought to apply our clustering approach in a second set of mIF (5-plex, Table A2) imaging data generated by an alternative platform (Polaris) (Figure 5a). To this end, tissue sections from two tonsils were used. Follicular areas were enriched in CD20 positive B cells (Figure 5a). Similar to the cycling mIF generated data (Figure 1b), PD-1^high^CD3^high^ Tfh cells were found exclusively expressed within the follicular areas (Figure 5a). We observed a low co-expression between CD3 and CD20 (Figure 5b). Our unsupervised clustering for the measured markers (CD20, CD3, PD-1, Ki67) showed showed that Ki67 was mainly associated with B cells while PD-1 was mainly co-expressed with CD3 (Figure 5c).

**Figure 6:**
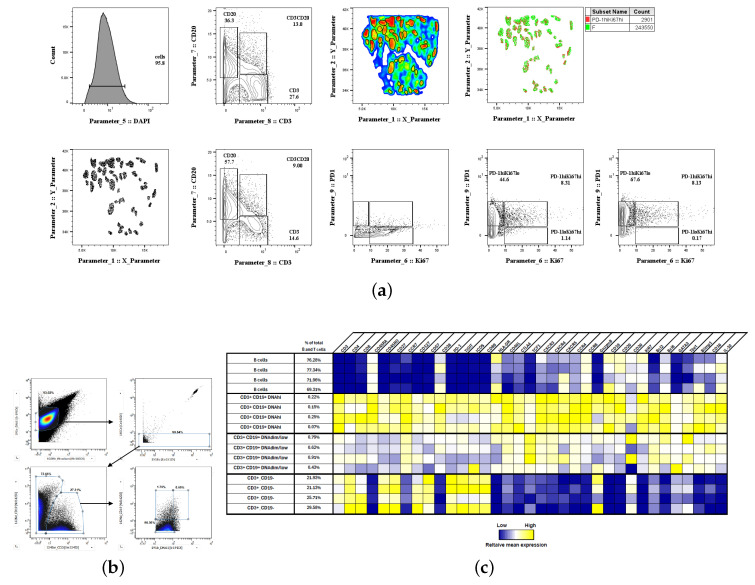
F/GCs represent areas with high densities of Tfh and B cells in close proximity. (**a**) mIF imaging data were analyzed by the HistoCytometry pipeline. The gating scheme for the identification of relevant immune cell subsets is shown. (**b**) CyTOF generated 2D plots showing the identified CD19 and CD3 cell subsets in a tonsillar single cell suspension (upper panel). Cell suspensions from four tonsils were used. (**c**) Clustering analysis showing the expression of individual molecules in CD19^high^ (B cells), CD3^high^CD19^low^ T cells as well as CD3^high^CD19^high^ T cells with high or dim/low DNA content (lower panel). A color bar index is also shown.

Then, imaging data (Polaris mIF) were analyzed with HistoCytometry, an established methodology for the analysis of imaging data using the FlowJo platform [14,31,32] (Figure 6a). Analysis of two tonsils showed a CD20^high^CD3^high^ cell population (9 and 11.5% of total follicular cells) (Figure 6a and Supplemental Figure S1) in line with the aforementioned identified clusters (Figure 3a). Although in lower frequency compared to CD20^low^CD3^high^, we found a high representation of CD3^high^PD-1^high^ Tfh cells in CD20^high^CD3^high^ cell compartment (Figure 6a and Supplemental Figure S1). In line with our unsupervised clustering analysis, Ki67 was found to be associated with B cells while PD-1 expression was almost exclusively associated with CD3 T cells (Figure 6a and Supplemental Figure S1).

### 3.2. Characterization of the Follicular CD3/CD20 ‘Conjugates’

We sought to further investigate the phenotype of the CD3/CD20 ‘conjugates’. To this end, the expression of several molecules in relevant tonsillar immune cell subsets was investigated in tissue-derived cell suspension with CyTOF (Figure 6b). The CD3/CD20 ‘conjugates’ were also evident in the CyTOF analysis (Figure 6b). Clustering analysis revealed a gradually increased expression of Tfh cell markers (e.g., PD-1, ICOS, TIGIT) between CD3^high^CD19^high^ DNA^dim/low^ and CD3^high^CD19^high^ DNA^high^ T cells (Figure 6c).

**Figure 7:**
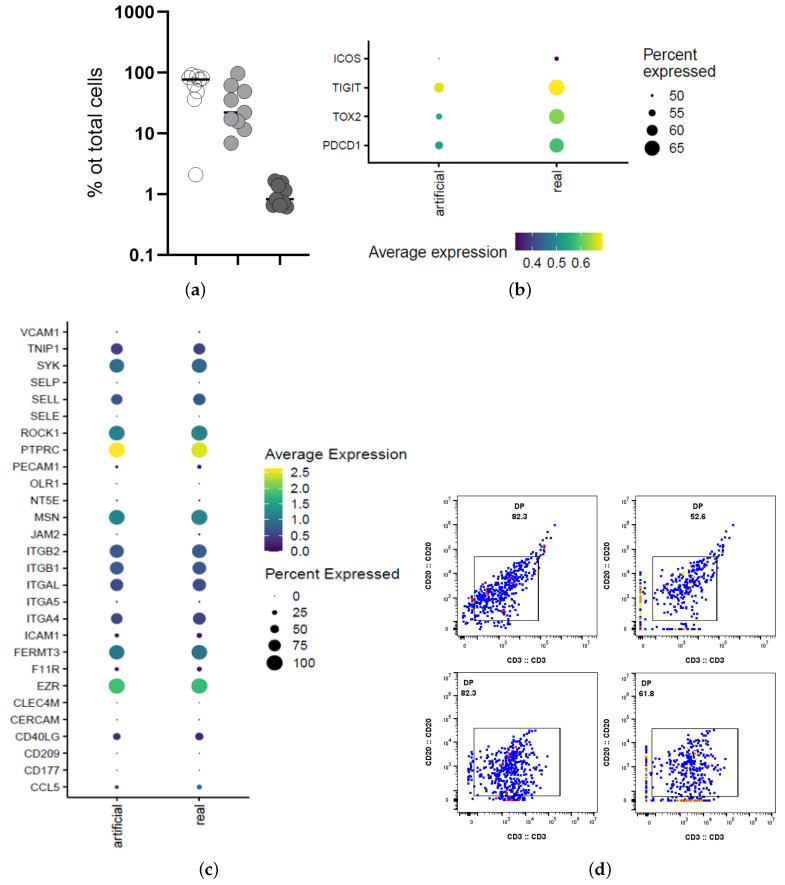
Transcriptomic characterization of CD3/CD20 ‘conjugates’. (**a**) accumulated data showing the relative frequency of CD20^high^CD3^low^ (white), CD20^low^CD3^high^ (light gray) and CD20^high^CD3^high^ (gray) in tonsils (n = 9). (**b**) the gene expression of Tfh cell biomarkers (PD-1, ICOS, TIGIT, TOX2) in the two types of CD3/CD20 ‘conjugates’ is shown. (**c**) dot plot showing the gene expression of leukocyte cell adhesion molecules in the two types of ‘conjugates’. (**d**) Flow cytometry 2D plots showing the co-expression of CD20 and CD3 proteins in one tonsillar (upper panel) and one LN (lower panel) follicular area before (left) and after (right) the application of the REDSEA algorithm.

We further characterized the molecular profile of the CD3/CD20 ‘conjugates’ using an online available tonsillar dataset (HCATonsilData). In line with the imaging and CyTOF data, ‘conjugates’ based on the co-expression of CD3 and CD20 genes we identified (Figure 7a). The comparative analysis of gene expression between in silico (artificial) CD3/CD20 ‘conjugates’ and the in situ detected ones (real), revealed a high expression of Tfh cell markers in the ‘real conjugates’ (Figure 7b). However, no significant difference was found between the two types of ‘conjugates’ when the gene expression for several adhesion molecules was investigated (Figure 7c). Application of the REDSEA [15] algorithm was able to further ‘clean’ the CD3/CD20 ‘conjugates’ (Figure 7d).

### 3.3. Unsupervised Clustering Analysis of Human Lymph Node Germinal Center Immune Cell Types

**Figure 8:**
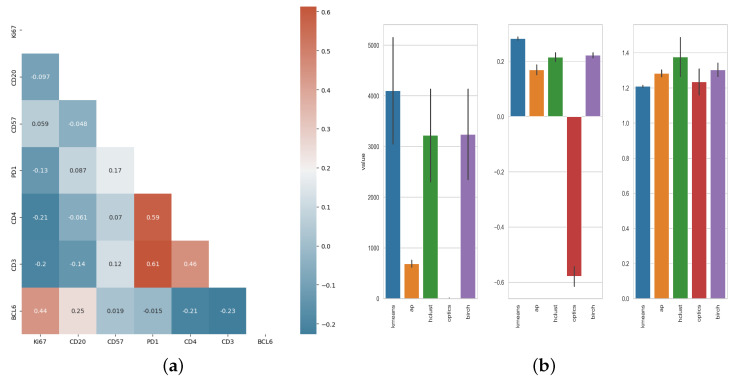
(**a**) Calculated correlations between markers used to characterize lymph node follicular/GC immune cell types using Pearson’s R. (**b**) Clustering scores for lymph nodes germinal center cell populations. Silhouette & Calinski-Harabasz: higher values indicate a better clustering result. Davies-Bouldin, lower values indicate a better clustering result.

Tonsils are a unique lymphoid organ for their anatomical site, structure and cellular composition. Despite the shared phenotypes between human tonsils and reactive lymph nodes [33] different immune dynamics are possible between the two organs, especially in the settings of a disease. We applied our approach to human lymph nodes characterized by follicular hyperplasia. Markers expression were found to be most correlated between PD1, CD3 and CD4 (Figure 8) similar to the results in Figure 3 and consistent with known cell phenotypes in GC. The Pearson’s R-value between CD57 and PD-1 however was noticeably lower for lymph nodes compared to tonsils.

**Figure 9:**
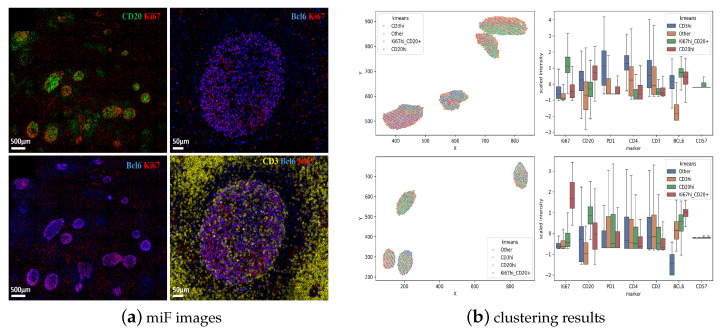
Results for follicular area in lymph nodes. (**a**) Example of expression of CD3, CD20, Bcl6 and Ki67, (**b**) Identification of immune cell clusters in lymph node follicular areas. Follicles/GCs from two reactive lymph nodes were used for our analysis. Coordinates-based representation of cell clusters produced with the Kmeans algorithm for both lymph nodes (left). Relative expression of individual analyzed markers in each immune cell cluster.

Similarly to the results for tonsils, the best-performing clustering algorithm for our lymph node samples was Kmean (Figure 8) with higher Silhouette and Calinski-Harabasz index and lower Davies-Bouldin index. This approach also identified clusters characterized by the co-expression of CD3, CD4, and PD-1 (Figure 9).

Bcl6 was mainly co-expressed with CD20, with or without Ki67. As expected, our approach showed that most follicular cells are B cells followed by CD3 T cells (Table 2). The cell population is smaller in lymph nodes’ germinal centers compared to the results of Table 1, mainly due to the difference in follicular/germinal center size between the two tissue types.

### 3.4. PD-1 Marker Prediction

**Figure 10:**
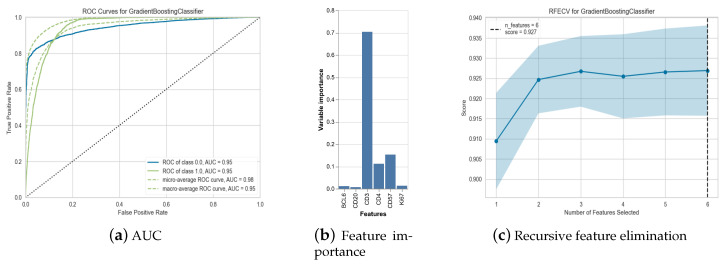
Model statistics: AUC curves for Gradient Boosting Classifier (best-performing model) with classes 0.0 = PD1 negative cells, classe 1.0= PD1 positive cells (**a**). Features importance for best performing model (**b**). RFE for the same model wit 95% confidence interval (**c**).

Then we asked whether mIF measured markers could predict the expression of PD-1. For the prediction of PD1 expression, among all models tested, the best performances were recorded for Gradient Boosting Classifier and Light Gradient Boosting Machine with AUC ranging between 0.9467 and 0.9456 (see Table 3 and Figure 10).

As expected from Figure 3 and Figure 8, the feature importance highlighted the predictive role of CD3 and CD4 for the best performing model (see Figure 10). This is in line with PD1 beeing expressed mainly by follicular T cells.

Recursive feature elimination (RFE) is shown in Figure 10. We can see that only 3 features are necessary to obtain our classification score with the model performance stagnating for additional variables. This suggests that cellular phenotype can be reliably inferred from very few markers.

## 4. Discussion

Given the complexity of the in situ operating cellular and molecular network under these conditions, mIF supported by unsupervised computational analysis tools is of great importance for the delineation of the immune landscaping and the possible impact of the disease on specific components of this network. As a starting point for the establishment of relevant pipelines, we have focused our analysis in well characterized anatomical sites (follicles/germinal centers) and associated immune phenotypes (Tfh, B cells) using a 8-plex mIF assay. We have successfully applied an unsupervised clustering method for analyzing mIHC data in pathology. Our results indicate a robust unsupervised clustering method using open-source methods and providing cellular marker expression consistent with known cell phenotypes. We found that among the explored unsupervised clustering algorithms Kmean provides the most robust performance. The superior performance of K-means in unsupervised clustering of cells based on MIF expression can be primarily attributed to its spherical cluster assumption and centroid-based approach. This methodology aligns remarkably well with the biological reality of cell populations expressing specific markers, which often exhibit a Gaussian distribution around a population mean. The identified cell clusters aligned well with known cell types in healthy germinal centers, including dark zone B cells, light zone B cells, and CD3/CD4 T cells. In addition to clustering analysis, we explored the ability of the generated set of imaging data to reliably predict the expression. of a given marker. Machine learning models, particularly Gradient Boosting Classifier and Light Gradient Boosting Machine, showed high accuracy in predicting PD1 expression based on other markers, indicating consistent marker expression.

Expansion (use of more biomarkers) and validation of the prediction value of a data set for specific molecules and for specific disease would be highly supportive for diagnostic purposes, especially if the capacity for performance of high dimension staining panels is limited. While these presented results are promising, several limitations should be addressed in future research. The lack of a gold standard for cellular clustering markers challenges the ultimate validation by comparing different clustering algorithms. The use of general-purpose clustering metrics and investigating the coherence of cell marker expression within cell populations through markers prediction can overcome, at least in part that limitation. However, more investigations using other metrics are needed to further assess the validity of those results. The suboptimal silhouette profiling for the clustering algorithm Kmean, could be attributed, at least in part, to noisy data and marker spillage between cells. We observed a ‘co-expression’ of T cell markers (CD3, CD4) by CD20^high^ B cells too. Several mechanisms could contribute to this profile. The closed proximity of T/B cells in the germinal center, especially the LZ, could lead to signal spillover between the two cell types [15]. This could potentially be avoided by focusing on nuclear markers for further studies. Data acquisition with higher resolution (e.g. use of 40X or 63X lens), application of algorithms such as REDSEA [15] and improvement of cell segmentation by using both cell membrane (e.g. use of anti-CD45RA) and nuclear staining could correct, at least in part, the detection of cell/cell conjugates. Interestingly, analysis of mIF data using the HistoCytometry platform, provided similar profiles with respect to CD3/CD20 ‘conjugates’ and the co-expression among CD3, CD20, PD-1 and Ki-67 further validating our approach. However, we should mention that part of the identified ‘conjugates’ may represent ongoing in vivo processes such as membrane ‘exchange’ between the two cell types, a process enhanced in SIV infection [34] and/or carrying of dead B blebs by Tfh cells [35]. Our CyTOF data, addressing relevant cell phenotypes in single cell suspension, suggests that part of these T/B cell ‘conjugates’ may represent stable Tfh/B cell interactions taking place in vivo [35], rather than an imaging artifact. Interestingly, the CD3^high^CD20^high^DNA^high^ cells are characterized by high expression of both Tfh and B cell markers. Furthermore, the transcriptomic analysis of public available data further confirmed the presence of these CD3/CD20 ‘conjugates’. Of note, these conjugates express relatively high levels of Tfh cell related markers. Further, the CD3/CD20 ‘conjugates’ express considerate levels of adhesion molecules (although not significantly different, there is a higher average expression of ICAM1 CD40L, CCL5 in the ‘real’ compared to ‘artificial’ conjugates) that could mediate, at least in part, the formation of the ‘conjugates’. Our in situ imaging analysis and the cell suspension analysis urge for further investigations of the possible role of these T/B cell conjugates as biomarkers for the pathogenesis of a disease like chronic HIV or SLE. We propose that a comprehensive analysis should take in account the calculation of these doublets together with the estimation of ’single cell’ data after the application of algorithms like REDSEA. Finally, the lack of an external independent dataset to validate the predictive capacity of our model for PD-1 expression is an important limitations in this part of our analysis. This should be evaluate with further investigations to access whether those results can be replicated. In conclusion, this study provides a robust framework for unsupervised analysis of mIHC data in pathology. It demonstrates the potential of combining advanced imaging techniques with machine learning algorithms to enhance our understanding of complex tissue microenvironments. However, for such a method to be introduced in a diagnostic pipeline, the generalization of these results should be confirmed by analyzing relevant data from different laboratories/Institutes.

## 5. Conclusions

This study demonstrates the successful application of an unsupervised clustering method for analyzing mIHC data in pathology. We found that amongst the explored unsupervised clustering algorithms K-mean provided the most robust performance. The identified cell clusters aligned well with known cell types in healthy germinal centers, including dark zone B cells, light zone B cells, and CD3+/CD4+ T cells. Supervised machine learning models, particularly boosting algorithms, showed high precision in predicting PD-1 expression based on other markers, indicating consistent marker expression. We should emphasize that this is a pilot study introducing alternative approaches for the analysis of imaging data. Given the importance of Tfh cells in chronic diseases like HIV, lymphomas, SLE, it is of great interest to develop computational tools that could improve their molecular characterization in order to better understand their role in disease pathogenesis as well as their in vivo manipulation in the context of an immunotherapy. Our data suggest that a ‘limited’ mIF assay (like the presented 8-plex assay) can reliably predict the cell density of Tfh cells in relevant LN tissues. Further, analysis of the spatial positioning (by applying algorithms for calculation of minimum distances among different cell tyoes) between germinla center B cells (proliferating or not) and Tfh cells could be applied. Following such an initial screening, further in situ deep phenotypic characterization of Tfh cells using more biomarkers (e.g. CXCR5, ICOS, TIGIT) should be applied to samples of interest. Given the availability of tissue-derived cells, the imaging data could be coupled to the characterization of Tfh and B cells and particularly the possible CD3/CD20 conjugates, at protein (CyTOF or flow cytometry) and/or transcriptomic (scRNA) level. The comparison between the prevalence of Tfh and B cells obtained by the different platforms could also indicate whether the imaged tissue level is representative of the whole tissue with respect to these cell subsets or not (‘sampling error’). Our complementary approach for the characterization of CD3/CD20 ‘conjugates’, where many of T cells are Tfh, could provide valuable information with respect to the biological significance of such conjugates which likely reflect actual in situ cellular cognate and non-cognate interactions. While these results are promising, several limitations should be addressed in future research: The lack of a gold standard for cellular clustering makes it challenging to definitively validate the results. The generalizability of the study is limited by the use of samples from a single institution. Suboptimal silhouette profiling suggests room for improvement in clustering accuracy, possibly through enhanced imaging techniques and marker expression analysis. In conclusion, this study provides a robust framework for unsupervised analysis of mIHC data in pathology. It demonstrates the potential of combining advanced imaging techniques with machine learning algorithms to enhance our understanding of complex tissue microenvironments. Future work should focus on validating these methods across multiple institutions and improving clustering accuracy to further advance the field of digital pathology.

## Figures and Tables

**Table 1:** Cell population clusters characterized by tonsil sample.

Sample	Num. GC ^1^	Clusters	Number of Cells
TS1	6	Ki67^high^CD20+	3841
		CD20^high^	3662
		CD3^high^	2239
		Other	1756
TS2	5	CD20^high^	13,419
		Other	10,244
		Ki67^high^CD20+	6831
		CD3^high^	6545

^1^ Number of GC used for analysis.

**Table 2:** Cell population clusters characterized by lymph nodes sample.

Sample	Num. GC ^1^	Clusters	Number of Cells
LN1	4	CD20^high^	2260
		Ki67^high^CD20+	1943
		CD3^high^	1347
LN2	4	CD20^high^	2395
		Other	1660
		CD3^high^	1519
		Ki67^high^CD20+	1153

^1^ Number of GC used for analysis.

**Table 3:** Models performance for the ten best-performing models in classifying of PD1 expression using lymph nodes and tonsils data.

Model	Accuracy	AUC	Recall	Prec.
Gradient Boosting Classifier	0.9269	0.9467	0.5297	0.6873
Light Gradient Boosting Machine	0.9263	0.9456	0.5251	0.6843
Ada Boost Classifier	0.9242	0.9425	0.5070	0.6787
Extreme Gradient Boosting	0.9236	0.9410	0.5157	0.6622
Extra Trees Classifier	0.9264	0.9405	0.4923	0.6991
Ridge Classifier	0.9208	0.9403	0.3400	0.7293
Linear Discriminant Analysis	0.9224	0.9403	0.5828	0.6307
SVM-Linear Kernel	0.9218	0.9395	0.4521	0.6958
Logistic Regression	0.9244	0.9394	0.4816	0.6825
Random Forest Classifier	0.9258	0.9390	0.4999	0.6919

## Data Availability

Code and preprocessed data used for this study can be found in our github repository: https://github.com/LTI-CHUV/clustering_MIF (accessed on 24 February 2025).

## Supplementary Materials

The following supporting information can be downloaded at: https://www.mdpi.com/article/10.3390/biology14050530/s1, Figure S1: HistoCytometry gating scheme for the identification of relevant cell types in the second tonsil imaged with the Polaris mIF platform.

## Abbreviations

The following abbreviations are used in this manuscript:

mIF	multiplex immunofluorescence
IHC	immunohistochemistry
LN	lymph node
TS	Tonsil
GC	germinal center
LZ	Light zone
DZ	Dark zone
Tfh	T-follicular helper cells

## Appendix A

**Table A1:** Demographic data for the used human lymph nodes.

Sample	Patient Age	Patient Gender	Localization
LN1	28	Female	inguinal
LN2	22	Female	cervical

## Appendix B

**Table A2:** Multiplex immunofluorescence assays used.

Specificity	Clone	Cat No	Conjugated/Unconjugated	Fluorophore	Cycle
**Cycling confocal mIF (8-plex)**					**2 cycles**
CD3	OTI3E10 (IgG2b)	TA506064	Unconjugated	Alexa-546 (Secondary)	1
CD4	Polyclonal	FAB8165N-100	Conjugated	Alexa-700	1
CD20	L26		Conjugated	eF615	1
Ki67	B56	561281	Conjugated	V450	1
Bcl6	GI191E/A8	760-4241	Unconjugated	Opal-690 (Secondary)	2
PD-1	Polyclonal	FAB7115G	Conjugated	Alexa-488	1
CD57	QA17A04	393326	Conjugated	BV421	1, 2
Mouse IgG2b	Polyclonal	A-21143	Conjugated	Alexa-546	1
Mouse IgG (OmniMap)	Polyclonal	760-4310	Conjugated	HRP substrate: Opal-690)	2
SYTO45		10297192			1, 2
**Polaris mIF (5-plex)**					**Order 1-cycle**
PD-1	NAT105	ACI 3137 AK, CK	Unconjugated	Opal 620	1
CD3	OTI3E10	TA506064	Unconjugated	Opal 570	2
CD20	L26	NCL-L-CD20-L26	Unconjugated	Opal 520	3
Ki67	MIB-1	M7240	Unconjugated	Opal 480	4
DAPI					5

**Table A3:** Mass Cytometry (CyTOF) phenotypic characterization of tonsillar-derived cells. Information for the antibodies used is shown.

Antibody	Metal	Clone	Company	Cat. N.	Amount
Cell Viability	103Rh	Cell-ID	Standard Biotools	201103A	0.6 μL
CD8	1123ln	RPA-T8	Biolegend	301018	0.6 μL
CD4	115ln	RPA-T4	Biolegend	300515	0.6 μL
CCR6	141Pr	11A49	Standard Biotools	3141014A	1 μL
CD19	142Nd	HIB19	Standard Biotools	3142001B	1.6 μL
CCR6	144Nd	G034E3	Standard Biotools	3141003A	1.88 μL
CD20	147Sm	2H7	Standard Biotools	3147001B	0.67 μL
ICOS	148Nd	C398.4A	Standard Biotools	3148019B	1 μL
CCR4	149Sm	L291H4	Standard Biotools	3149029A	1.5 μL
CD40L	152Sm	TRAP1	BD Biosciences	555698	3 μL
TIGIT	153Eu	MBSA43	Standard Biotools	3153019B	2 μL
CD3	154SM	UCHT1	Standard Biotools	3154003B	0.5 μL
CD27	155Gd	L128	Standard Biotools	3155001B	1 μL
CXCR3	156Gd	G025H7	Standard Biotools	3156004B	0.8 μL
CCR7	159Td	G043H7	Standard Biotools	3159003B	1 μL
CD30	162Dy	BY88	Biolegend	333902	3 μL
CXCR5	164Dy	RF8B2	Standard Biotools	3164029B	1.2 μL
CD45RO	165Ho	UCHL1	Standard Biotools	3165011B	0.7 μL
CD38	167Er	HIT2	Standard Biotools	3167001B	0.6 μL
CD45RA	170Er	HI100	Standard Biotools	3171001B	1.6 μL
HLA-DR	174Yb	L243	Standard Biotools	3174001B	0.7 μL
CD279/PD-1	175Lu	EH12.2H7	Standard Biotools	3175008B	1 μL
CD127	176Yb	A019D5	Standard Biotools	3176004B	0.7 μL
CTLA4	174Pt	Ipilimumab	BMS	ACB3065	2.5 μL
CD57	198Pt	NK-1	BD Biosciences	555618	1 μL
CD16	209Bi	3G8	Standard Biotools	3209002B	1.5 μL
Anti-PE	145Nd	PE001	Standard Biotools	3145006B	2 μL
GATA-3	146Nd	TWAJ	ThermoFisher	14-9966-82	1 μL
Tbet	161Dy	4B10	Standard Biotools	3161015B	2 μL
Bcl6	163Dy	K112-91	Standard Biotools	3163012B	2 μL
Ki67	168Tm	Ki-67	Standard Biotools	3168001B	1.4 μL
Blimp1	169Tm	646702	Bio-techne	MAB36081	3 μL
Granzyme B	171Yb	GB11	Standard Biotools	3171002B	2 μL
Bcl2	173Yb	100	Biolegend	658702	2 μL
