# Supplementary figures and images for: Unsupervised Clustering of Cell Populations in Germinal Centers Using Multiplexed Immunofluorescence
Source: Biology (Basel). 2025 May 11;14(5):530. doi: 10.3390/biology14050530 (PMC12108741; doi:10.3390/biology14050530)

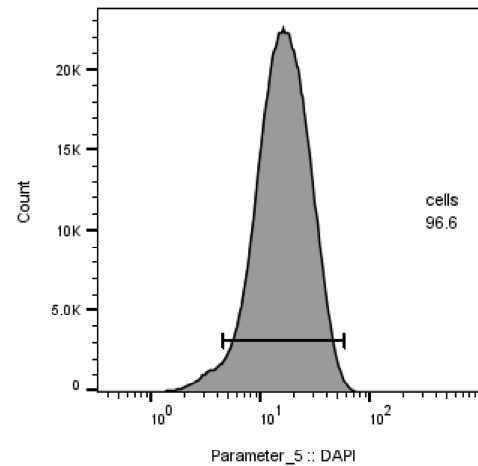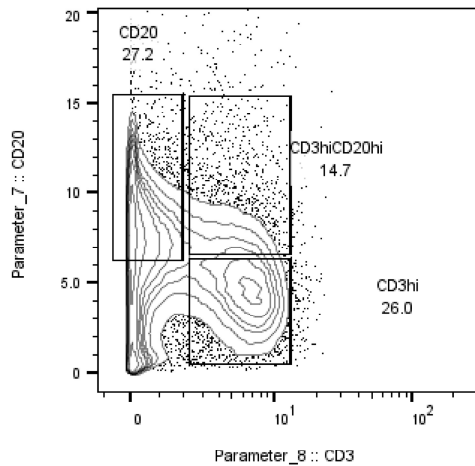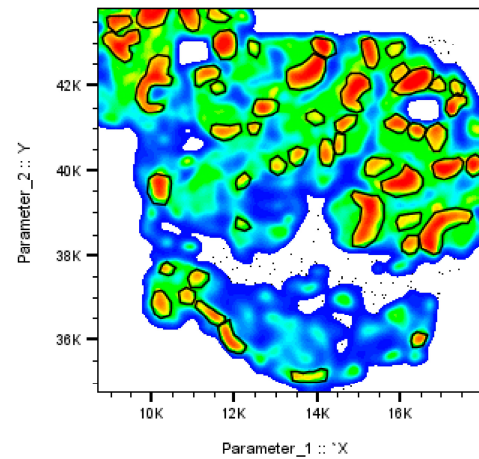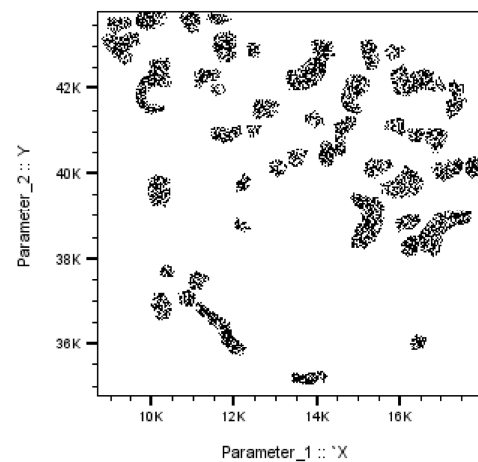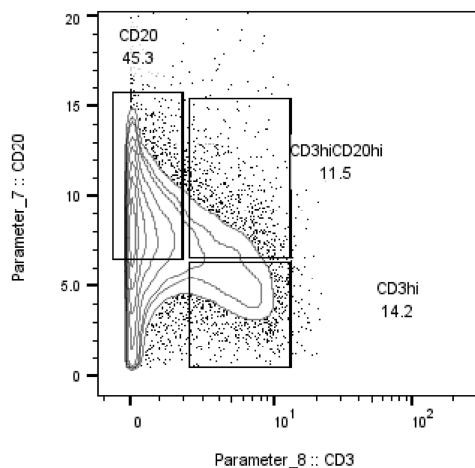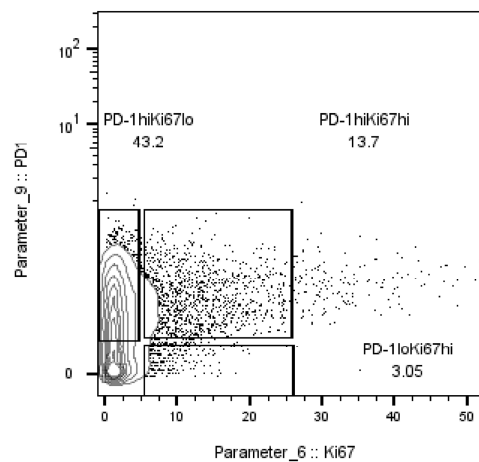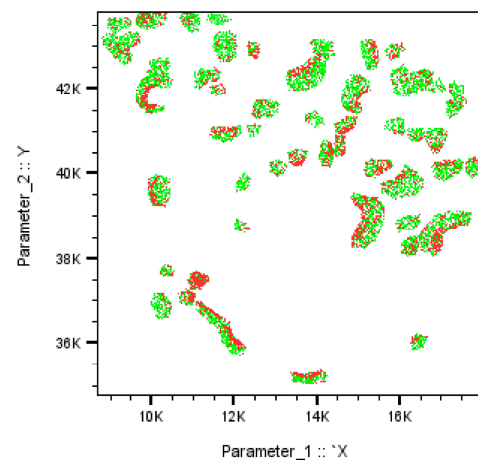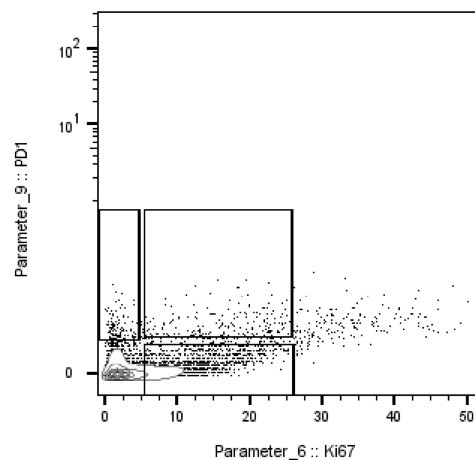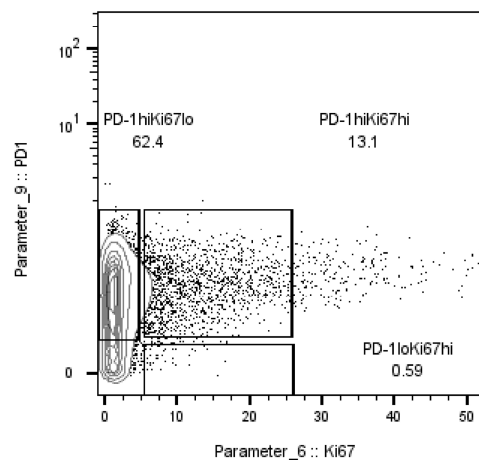

|   | Subset Name  | Count  |
|---|--------------|--------|
| ■ | PD-1hiKi67hi | 4178   |
| ■ | F            | 225029 |

Supplement: Supplementary file 1 [file biology-14-00530-s001.zip › biology-3529544-supplementary.pdf]
